# Center-Related Variation in Hospitalization Cost for Patients Undergoing Percutaneous Left Atrial Appendage Occlusion

**DOI:** 10.1016/j.shj.2024.100376

**Published:** 2024-10-24

**Authors:** Shivaraj Patil, Chaitanya Rojulpote, Abhijit Bhattaru, Avica Atri, Krishna Vamsi Rojulpote, Ola Khraisha, Viha Atri, William Frick, Tarek Nafee, Kishore Harjai, Sumeet Mainigi, Chien-Jung Lin

**Affiliations:** a Division of Cardiology, Department of Medicine, Einstein Medical Center, Philadelphia, Pennsylvania, USA; b Division of Cardiology, Department of Medicine, Saint Louis University, Saint Louis, Missouri, USA; c Division of Cardiovascular Imaging, Department of Medicine, Hospital of the University of Pennsylvania, Pennsylvania, USA; d Division of Medicine, Loyola University Chicago Stritch School of Medicine, Maywood, Chicago, Illinois, USA; e Department of Medicine, Kasturba Medical College, Manipal, Karnataka, India

**Keywords:** Left atrial appendage occlusion, Atrial fibrillation, Cost variation, Watchman device

## Abstract

**Background:**

The commercial use of percutaneous left atrial appendage occlusion with the Watchman device is increasing in the United States. The purpose of this study was to evaluate center-related variation in total hospital costs for Watchman device implantation and identify factors associated with high hospital costs at a national level.

**Methods:**

All adults undergoing elective left atrial appendage occlusion with Watchman were identified in the 2016-2018 National Inpatient Database. Mixed models were used to evaluate the impact of center on total hospital costs, adjusting for patient and center characteristics and length of stay.

**Results:**

A total of 30,175 patients underwent Watchman device implantation at a median cost of $24,500 and demonstrated significant variability across admissions (interdecile range, $13,900-37,000). Nearly 13% of the variability in patient-level costs was related to the center performing the procedure rather than patient factors. Higher-volume centers had lower total costs and demonstrated lesser total cost variation. Centers with low procedural volume, occurrence of procedural complications, congestive heart failure, and length of stay were independent predictors of a high-cost hospitalization. Though complications were associated with increased expenditure, they did not explain the observed cost variation related to the center.

**Conclusions:**

A significant proportion of variation in total hospital cost was attributable to the center performing the procedure. Addressing variability of Watchman-related costs is necessary to achieve high-quality value-based care.

## Key Highlights

1. What is already known:
  • Watchman device implantation is a cost-effective alternative for stroke prevention in atrial fibrillation patients unsuitable for long-term oral anticoagulation therapies.
2. What this study adds:
  • There are significant differences in costs between hospitals for the Watchman device procedure, highlighting the need for standardizing hospital practices and optimizing costs.
  • Variation in hospital costs for the Watchman device procedure is influenced by differences in hospital care pathways and resource utilization, with factors such as low procedural volume, procedural complications, congestive heart failure, and longer hospital stays contributing to increased costs.
3. How this study might affect research, practice, or policy:
  • Our findings demonstrate considerable variability in patient costs for elective left atrial appendage occlusion attributable to the hospital and emphasize the need for further studies analyzing cost variation related to implantation of more contemporary left atrial appendage occlusion devices to develop targeted cost containment strategies.

## Introduction

Stroke among atrial fibrillation (AF) patients is a major cause of disability and substantial economic burden.^1^ Long-term oral anticoagulation (OAC) has been the mainstay of treatment for prevention of cardioembolic stroke in AF. Percutaneous left atrial appendage occlusion (LAAO) has become an attractive alternative to reduce the risk of stroke in patients with nonvalvular AF when OAC is not suitable or hazardous.^2^

LAAO with the Watchman device (Boston Scientific, Marlborough, Massachusetts) was approved in March 2015 in the United States and is being increasingly utilized for mitigation of thromboembolic risk.^3^ While the initial cost of LAAO with the Watchman device is high, it has proven to be a cost-effective treatment strategy compared to vitamin K antagonists and non-vitamin K antagonist OAC therapies.^4^^,^^5^ With the sustained rise in US health care expenditures and increasing emphasis on value-based health care delivery, examination of costs associated with Watchman device implantation is particularly relevant.^6^

To date, variation in Watchman procedure-related expenditures has not been studied. Thus, the purpose of this study was to evaluate center-related variation in total hospital costs for Watchman device implantation and identify factors associated with high hospital costs at a national level.

## Methods

### Data Source

We performed a 3-year population-based retrospective cross-sectional analysis using national (United States) data from January 2016 to December 2018. The National Inpatient Sample (NIS) database is the largest publicly available all-payer inpatient care database from the United States. It is developed as a part of the Healthcare Cost and Utilization Project (HCUP) and is sponsored by the Agency for Healthcare Research and Quality, available at https://www.hcup-us.ahrq.gov/overview.jsp. The NIS includes data from all nonfederal, short-term, general, and other specialty hospitals in the United States (excluding rehabilitation and long-term acute care hospitals) in the form of deidentified patient information containing demographics, discharge diagnoses, comorbidities, procedures, outcomes, and hospitalization costs. All states that participate in HCUP provide data to the NIS, covering >95% of the US population. The database was designed to include data from a 20% sample of discharges from all participating hospitals. This design of the NIS reduces the margin of error for estimates and delivers more stable and precise estimations. The study was exempt from an institutional review board approval because HCUP-NIS is a publicly available database containing only deidentified patient information.

### Study Population

All adults (age ≥18 years) who underwent elective LAAO with Watchman device were identified using the International Classification of Diseases-10th Revision procedure code 02L73DK. Patients with missing data on age, sex, hospitalization costs, and in-hospital mortality were excluded. Furthermore, to reduce the possibility of data duplication, patients with an indicator for transfer to another acute-care facility were excluded.

### Variable Definitions

Baseline patient characteristics including age, sex, race, income level, and payer status were defined in accordance with the NIS data dictionary. The previously validated Charlson comorbidity index (CCI) was used to quantify the burden of chronic conditions. In-hospital major adverse events (MAEs) were defined as the composite of mortality, stroke (ischemic or hemorrhagic) or transient ischemic attack, bleeding or transfusion, vascular complications, myocardial infarction, systemic embolization, and pericardial effusion or tamponade requiring pericardiocentesis or surgery. The International Classification of Diseases-10th Revision codes used to define these variables are listed in Supplemental Table 1. Annual hospital volume was calculated as the total number of elective Watchman device implantation performed at each center. Hospitals were subsequently classified into low volume: ≤15 procedures/year (LVH), medium volume: 16-35 procedures/year (MVH), and high volume: ≥36 procedures/year (HVH) based on their annual case load. Hospitalization costs were generated by application of a hospital-specific cost-to-charge ratio and inflation adjusted to 2018. Total hospitalization costs represent the expenses incurred in the production of hospital services, such as wages, supplies, and utility. However, physician professional fees are not captured by the NIS database. Admission was designated as a high-cost hospitalization if total unadjusted hospitalization cost was in the highest decile.

### Outcome

The primary outcome was total hospitalization cost at patient level and its variation related to center-level differences. We also analyzed the variation in MAE attributable to center-level differences due to high correlation between the incidence of complications and hospitalization costs at the patient level. Secondarily, we assessed patient characteristics and predictors of high-cost hospitalization for LAAO with Watchman device.

### Statistical Analysis

National estimates were calculated by applying discharge weights. Categorical variables are reported as proportions and compared using Pearson’s chi-squared test. Continuous variables are reported as means with SD or median with interquartile range (IQR), when appropriate. Means and medians were compared using independent samples t-test and Mann-Whitney U test, respectively. Median costs from 2016 to 2018 and between LVH, MVH, and HVH were compared using nonparametric, independent samples Kruskal-Wallis test. A multivariate regression model with high-cost hospitalization status as dependent variable was developed to examine predictive factors. To evaluate the effect of individual center on total hospital costs, a 2-level log-gamma generalized mixed effects model was used with centers as a random effect because of the skewed distribution of cost data (Supplementary figures 1-5). The proportion of total cost variation explained by the random center effect was calculated. SPSS Statistics 25.0 (IBM Corp., Armonk, New York) and R statistical software (R Core Team 2020) were used to perform the statistical analysis. All *p* values were 2-sided with a significance threshold of <0.05.

## Results

**Figure 1:**
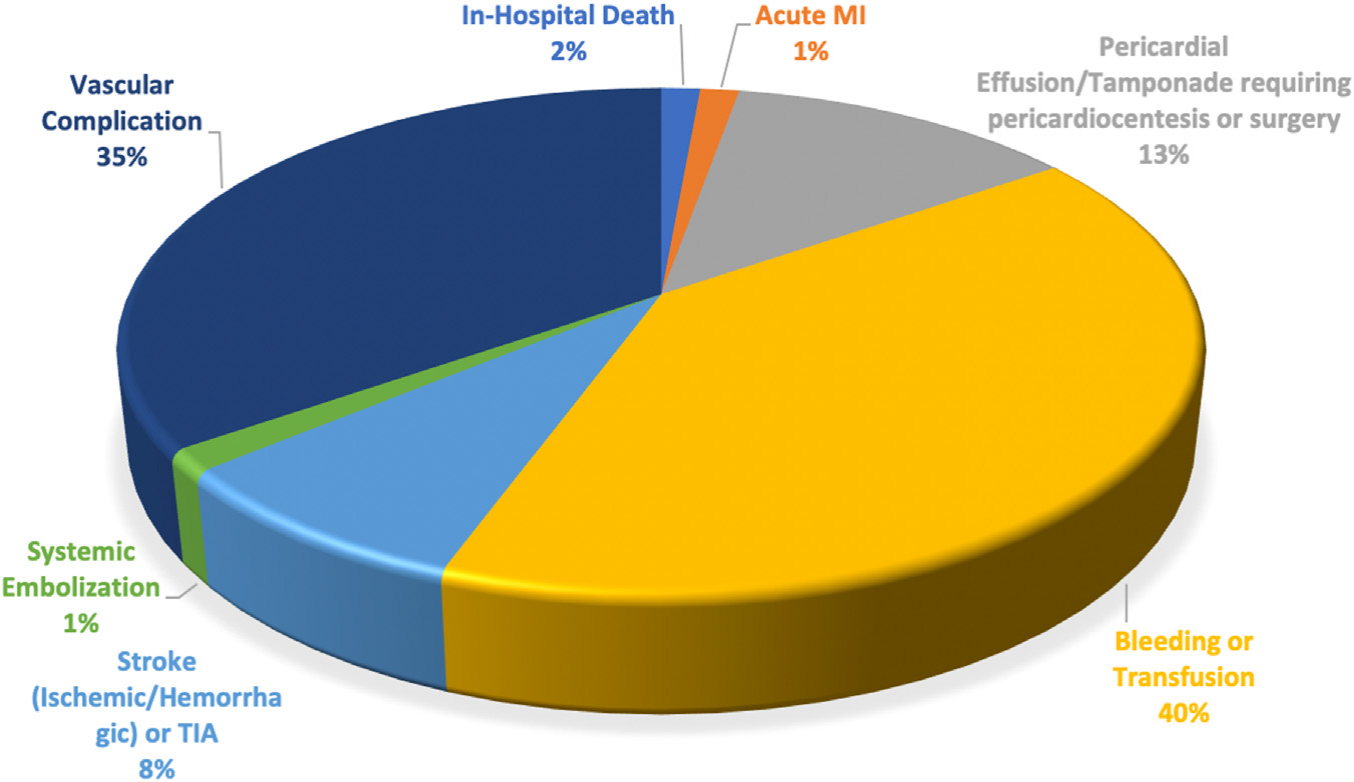
Distribution of in-hospital major adverse events related to Watchman device implantation. Abbreviations: MI, myocardial infarction; TIA, transient ischemic attack

A total of 30,175 patients met the study criteria and underwent elective admission for Watchman device implantation at an average of 290 hospitals per year across the United States. The mean age was 76 years, and women constituted 41.7% of the cohort. Less than 20% of the patients were in the highest income quartile, and Medicare was the primary insurer for most patients (89%). Congestive heart failure was the most common comorbidity, and median CCI score was 1 [1-3]. The vast majority of patients (62.5%) underwent Watchman device implantation at a high-volume hospital. A MAE occurred in 4.6% of the study cohort with bleeding/transfusion (2.9%) and vascular complication (2.5%) being the most common events. In-hospital mortality was 0.1% (Table 1, Figure 1). The rates of MAEs were lower in HVH compared to LVH (4.3 vs. 5.1%, *p* = 0.016).

**Table 1:** Baseline characteristics

Variable	Count	Summary statistic
Age (y, mean ​± ​SD)	-	76.02 ​± ​7.97
Female (%)	12,585	41.7
White (%)	25,375	84.1
Charlson comorbidity index (median, IQR)	-	1 [1-3]
Comorbidities (%)		
Congestive heart failure	11,595	38.4
Coronary artery disease	3755	12.4
Peripheral vascular disease	4930	16.3
Cerebrovascular disease	2275	7.5
Chronic obstructive pulmonary disease	6605	21.9
Chronic kidney disease	7220	23.9
Moderate-severe liver disease	155	0.5
Diabetes	10,455	34.6
Income quartile		
76th-100th	7585	19.8
51st-75th	8390	25.8
26th-50th	7780	27.8
1st-25th	5980	25.1
Payer status		
Medicare	26,850	89.0
Medicaid	340	1.1
Private	2370	7.9
Other	545	1.8
Hospital region		
New England	820	2.7
Middle Atlantic	3720	12.3
East North Central	4255	14.1
West North Central	2475	8.2
South Atlantic	6345	21
East South Central	1725	5.7
West South Central	3660	12.1
Mountain	3025	10
Pacific	4150	13.8
Hospital volume		
Low volume (1-15 procedures/year)	3360	11.1
Medium volume (16-35 procedures/year)	7955	26.4
High volume (>35 procedures/year)	18,860	62.5
Major adverse event (%)	1385	4.6
In-hospital death	40	0.1
Acute MI	20	0.1
Pericardial effusion/tamponade requiring pericardiocentesis or surgery	285	0.9
Bleeding or transfusion	885	2.9
Stroke (ischemic/hemorrhagic) orTIA	175	0.6
Systemic embolization	30	0.1
Vascular complication	755	2.5
Median length of stay (d)		1
Median cost (interdecile range)		$24,500 ($13,900-37,000)

Abbreviations: IQR, interquartile range; MI, myocardial infarction; TIA, transient ischemic attack.

On a national level, the median unadjusted patient-level hospitalization cost for Watchman device implantation was $24,500 and demonstrated significant variability across admissions in our study cohort (interdecile range, $13,900-37,000). The median hospitalization costs decreased slightly over the study period from $24,600 [IQR, $18,900-30,900] in 2016 to $24,400 [IQR, $18,600-29,800] in 2018 (*p* ​< ​0.001). As expected, patient-level costs were significantly (*p* ​< ​0.001) greater for patients experiencing a MAE: $28,700 (IQR, $21,700-37,100) compared to those who did not experience a MAE: $24,400 (IQR, $18,600-30,100). Median hospitalization costs were significantly lower in HVH: $24,000 (IQR, $18,700-29,200), compared to LVH: $25,900 (IQR, $20,100-33,400) in our study sample (*p* < ​0.001). Additionally, there was significant variation (*p* < 0.001) in median hospitalization costs based on primary payer: Medicare: $24,600 (IQR, $19,000-30,400), Medicaid: $24,900 (IQR, $19,800-30,900), private pay: $24,900 (IQR, $18,000-30,400), and other pay: $17,100 (IQR, $8800-27,900).

**Figure 2:**
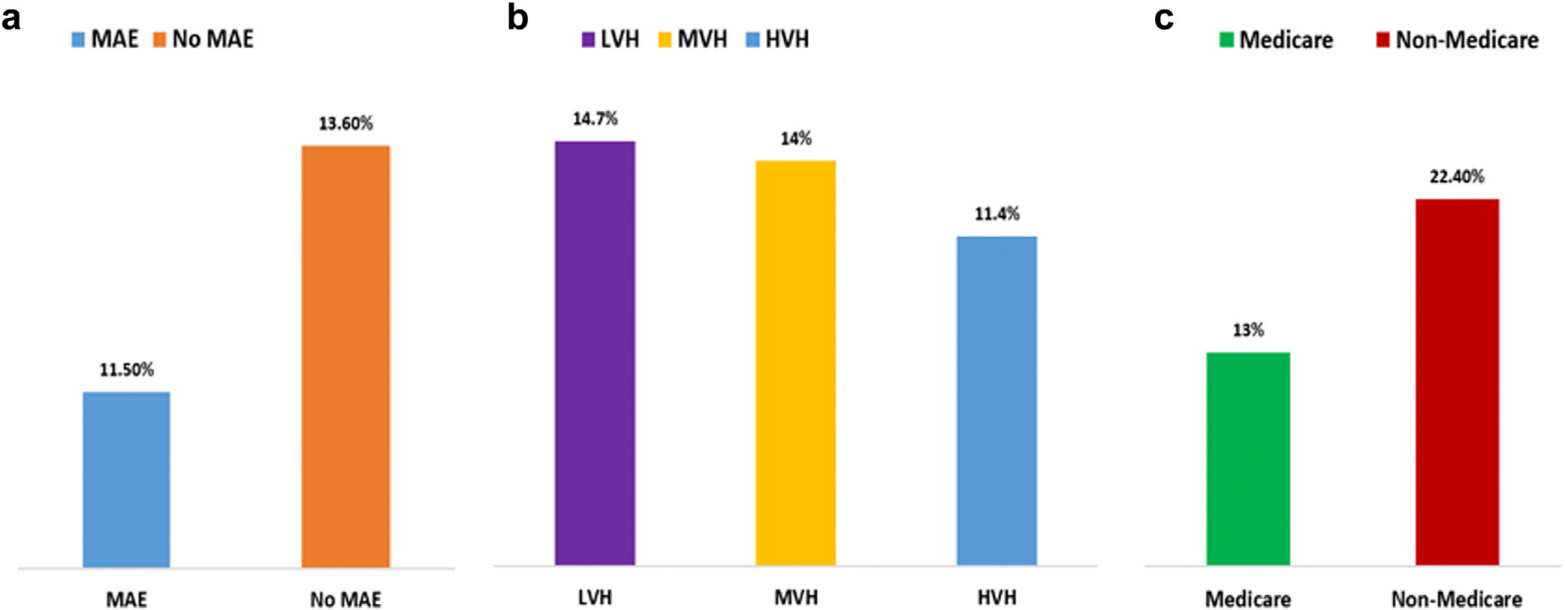
Degree of interhospital variation in cost following Watchman implantation. Panel (a) shows the degree of variation among patients with and without major adverse events. Panel (b) shows the degree of variation based on hospital Watchman implantation volume. Panel (c) shows the degree of variation in Medicare and non-Medicare beneficiaries. Abbreviations: HVH, high volume hospital; LVH, low volume hospital; MAE, major adverse event; MVH, medium volume hospital.

Analysis of random intercept from the mixed model revealed 13.3% (95% CI: 12.1%-14.7%) of total cost variation for Watchman device implantation was due to the center-level differences. Among patients who experienced a MAE, 11.5% (95% CI: 9.6%-13.8%) of interhospital variation in total costs was attributable to center level differences, compared to 13.6% (95% CI: 12.3%-15%) in those who did not experience a MAE. On examining the relationship between annual hospital volume for Watchman device implantation and cost variation, we observed a decline in the proportion of cost variation attributable to the center with 14.7% in LVH (95% CI: 12.6%-17.2%), 14% (95% CI: 11.9%-16.3%) in MVH, compared to 11.4% (95% CI: 9.7%-13.5%) in HVH. For patients primarily insured by Medicare, 13% (95% CI: 11.8%-14.3%) of interhospital variation of total cost was attributable to center-level differences, while this variation was 22.4% (95% CI: 19.6%-26.5%) among non-Medicare (Medicaid, private pay, other) patients (Figure 2, Table 2). Notably, White race (coef: −0.034, SE: 0.008; *p* ​< ​ 0.001) and lower CCI score: 3-4 (coef: - 0.038, standard error: 0.015; *p* ​= ​0.014) were statistically associated with lower costs.

**Table 2:** Factors associated with total hospital costs in generalized mixed effects model

Variable	Coefficient estimate	Standard error	*P* value
Age	−0.001	0.0	0.109
Female	9.67	0.035	<0.001
White	−0.034	0.008	<0.001
Income quartile			
0-25th	−0.007	0.008	0.342
26-50th	0.022	0.007	0.002
51-75th	0.017	0.007	0.011
76-100th	Reference	-	-
Payer status			
Medicare	0.339	0.018	<0.001
Medicaid	0.317	0.029	<0.001
Private	0.225	0.02	<0.001
Other	Reference	-	-
Hospital volume			
Low volume center	0.042	0.02	0.04
Medium volume center	0.083	0.017	<0.001
High volume center	Reference	-	-
Major adverse event	0.096	0.012	<0.001
Comorbidities			
Coronary artery disease	0.017	0.008	0.036
Congestive heart failure	0.052	0.006	<0.001
Peripheral vascular disease	0.024	0.007	0.001
Cerebrovascular disease	−0.003	0.01	0.78
COPD	0.008	0.007	0.22
Diabetes	0.014	0.007	0.038
Chronic kidney disease	−0.012	0.007	0.092
Moderate-severe liver disease	0.109	0.034	0.001
Charlson comorbidity index score			
CCI ≥ 5	−0.033	0.025	0.2
CCI ​= ​3-4	−0.038	0.015	0.014
CCI ​= 1-2	−0.01	0.008	0.22
CCI ​= ​0	Reference	-	-
Length of Stay	0.66	0.002	<0.001

Abbreviation: CCI, Charlson comorbidity index; COPD, chronic obstructive pulmonary disease.

Admissions with total unadjusted hospitalization costs >90th percentile ($37,000) were categorized as high-cost hospitalizations. Compared with others, patients who experienced a high-cost hospitalization had similar distribution of age and primary payer status (Medicare) (Table 3). High-cost hospitalization patients were more commonly women, and approximately 30% belonged to the highest income quartile. The burden of congestive heart failure and MAEs were significantly greater among high-cost hospitalizations. Among patients who did not experience a high-cost hospitalization, nearly 64% of patients underwent Watchman device implantation at a HVH, and only 11% of patients were treated at a LVH. Whereas, patients who experienced a high-cost hospitalization, a greater proportion of patients were treated at a LVH (15.9%), and only 52% were treated at a HVH (Table 4).

**Table 3:** Comparison of baseline characteristics between high-cost and nonhigh-cost hospitalization

Variable	Nonhigh-cost hospitalization	High-cost hospitalization	*P* value
Age (y, mean ​± ​SD)	76.03 ​± ​7.98	75.89 ​± ​7.84	0.34
Female (%)	11,265 (41.3)	1320 (45.7)	<0.001
White (%)	84	84.8	0.2
Charlson comorbidity index (median, IQR)	1 [1-3]	1 [1-3]	0.018
Comorbidities (%)			
Congestive heart failure	12.6	11.2	0.001
Coronary artery disease	38.1	41.2	0.04
Peripheral vascular disease	16.5	14.5	0.006
Cerebrovascular disease	7.6	7.3	0.56
Chronic obstructive pulmonary disease	21.9	21.6	0.72
Chronic kidney disease	24.2	20.9	<0.001
Moderate-severe liver disease	0.5	0.7	0.16
Diabetes	34.8	32.9	0.035
Income quartile (%)			
76th-100th	24.6	29.8	0.001
51st-75th	28	26	0.024
26th-50th	25.9	24.4	0.087
1st-25th	20.0	18.2	0.023
Payer status (%)			
Medicare	88.9	90.1	0.083
Medicaid	1.2	0.7	0.019
Private	7.9	8.0	0.857
Other	1.9	1.2	0.011
Hospital region			<0.001
New England	2.5	4.5	
Middle Atlantic	12.2	13.7	
East North Central	14.6	9.3	
West North Central	8.5	5	
South Atlantic	21.2	19.4	
East South Central	5.5	7.6	
West South Central	12	13	
Mountain	10	9.9	
Pacific	13.3	17.6	
Hospital volume			<0.001
Low volume (1-15 procedures/year)	10.6	15.9	
Medium volume (16-35 procedures/y)	25.7	32.5	
High volume (>35 procedures/y)	63.7	51.6	
Major adverse event (%)	1060 (3.9)	325 (11.2)	<0.001
In-hospital death	15 (0.1)	25 (0.9)	<0.001
Acute MI	20 (0.1)	-	
Pericardial effusion/tamponade requiring pericardiocentesis or surgery	150 (0.5)	130 (4.7)	<0.001
Bleeding or transfusion	655 (2.4)	230 (8.0)	<0.001
Stroke (ischemic/hemorrhagic) or TIA	170 (0.6)	<10 (0.2)	0.002
Systemic embolization	25 (0.1)	<10 (0.2)	0.182
Vascular complication	565 (2.1)	190 (6.6)	<0.001
Median length of stay (IQR)	1 (1)	1 (1-2)	<0.001
Median cost (IQR)	$23,600 ($18,100-28,400)	$43,500 ($39,500-51,300)	<0.001

Abbreviations: IQR, interquartile range; MI, myocardial infarction; TIA, transient ischemic attack.

**Table 4:** Comparison of procedural volume between high-cost and nonhigh-cost hospitalization

Variable	Nonhigh-cost hospitalization	High-cost hospitalization	*P* value
Hospital volume			<0.001
Low volume (1-15 procedures/y)	10.6	15.9	
Medium volume (16-35 procedures/y)	25.7	32.5	
High volume (>35 procedures/y)	63.7	51.6	
Hospital region			<0.001
New England	2.5	4.5	
Middle Atlantic	12.2	13.7	
East North Central	14.6	9.3	
West North Central	8.5	5	
South Atlantic	21.2	19.4	
East South Central	5.5	7.6	
West South Central	12	13	
Mountain	10	9.9	
Pacific	13.3	17.6	

On multivariate analysis, lower procedural volume center (adjusted odds ratio [aOR]: 2.11, 95% CI: 1.875-2.376; *p* ​= 0.001), occurrence of MAE (aOR: 1.281, 95% CI: 1.073-1.53; *p* ​= 0.006), congestive heart failure (aOR: 1.25, 95% CI: 1.072-1.459; *p* ​= 0.004), high comorbidity burden (CCI ​= ​6) (aOR: 2.504, 95% CI: 1.122-5.59; *p* = 0.025), and length of stay (aOR: 1.412, 95% CI: 1.369-1.458; *p* = 0.001) were independent predictors of a high-cost hospitalization (Table 5).

**Table 5:** Multivariate regression model to predict factors associated with high cost hospitalization

Variable	aOR	95% CI	*P* value
Age	0.99	0.985-0.996	0.001
Female	1.05	0.966-1.142	0.25
White	0.921	0.815-1.04	0.182
Income quartile			
0-25th	0.793	0.7-0.899	0.001
26-50th	0.83	0.742-0.93	0.001
51-75th	0.721	0.646-0.805	0.001
76-100th	Ref		
Payer status			
Medicare	1.309	0.922-1.858	0.133
Medicaid	0.373	0.195-0.715	0.003
Private	1.119	0.767-1.633	0.559
Other	Ref		
Hospital region			
New England	0.612	0.491-0.763	0.001
Middle Atlantic	0.336	0.266-0.426	0.001
East North Central	0.374	0.288-0.487	0.001
West North Central	0.506	0.407-0.629	0.001
South Atlantic	0.9	0.702-1.155	0.408
East South Central	0.626	0.498-0.786	0.001
West South Central	0.578	0.455-0.733	0.001
Mountain	0.811	0.653-1.007	0.058
Pacific	Ref		
Hospital volume			
Low volume center	2.11	1.875-2.376	0.001
Medium volume center	1.694	1.544-1.858	0.001
High volume center	Ref		
Major adverse event	1.281	1.073-1.53	0.006
Comorbidities			
Coronary artery disease	0.958	0.8-1.147	0.639
Congestive heart failure	1.25	1.072-1.459	0.004
Peripheral vascular disease	0.9	0.761-1.064	0.218
Cerebrovascular disease	0.828	0.675-1.016	0.071
COPD	0.92	0.784-1.079	0.305
Diabetes	0.932	0.797-1.091	0.384
Chronic kidney disease	0.792	0.67-0.936	0.006
Moderate-severe liver disease	0.988	0.55-1.774	0.966
CCI score			
CCI score ​= 1	0.931	0.79 -1.098	0.396
CCI score ​= 2	0.865	0.659-1.135	0.295
CCI score ​= ​3	0.852	0.578-1.256	0.418
CCI score ​= ​4	0.714	0.43-1.188	0.195
CCI score ​= 5	0.571	0.287-1.138	0.111
CCI score ​= ​6	2.504	1.122-5.59	0.025
Length of stay	1.412	1.369-1.458	0.001

Abbreviation: aOR, adjusted odds ratio; CCI, Charlson comorbidity index; COPD, chronic obstructive pulmonary disease.

## Discussion

In this large nationwide study of patients undergoing LAAO with the Watchman device, we found significant variation in total hospitalization costs. Nearly 13% of the variability in patient-level costs was related to the center performing the procedure. These differences persisted even after adjustment for patient characteristics (demographics, comorbidities, insurance), procedural complications, hospital location, hospital volume, and length of stay. Similarly, among patients experiencing a major adverse event, substantial variation in costs attributable to the center was observed. Interestingly in our study cohort, less than 1% of the variation in complications was attributable to interhospital differences. Taken together, our findings suggest that differences in hospital care pathways and resource utilization, rather than the incidence of procedure-related complications, were major drivers for the observed center-level variation in costs for Watchman device implantation. For instance, a center (or operator) may prefer implanting the Watchman device under general anesthesia, tracheal intubation, and a dedicated intraprocedural echocardiographer for transesophageal echocardiographic guidance, followed by routine overnight inpatient observation, whereas another center (or operator) may perform this procedure with conscious sedation, intracardiac echocardiography, and same-day discharge if deemed appropriate. Therefore, minimalistic, effective, and safe strategies employed by centers for elective Watchman implantation could result in reduced patient costs.

These interhospital differences in total patient costs were also more prominent for patients undergoing Watchman device implantation at low-volume centers compared to high-volume centers. This could possibly be due to greater operator experience, lower rates of complications, greater availability of services, and utilization of standardized protocols of care at high-volume centers.^7^^,^^8^

Despite the proliferation of value-based health care in the United States, variation in costs across different hospitals among Medicare beneficiaries has been previously reported.^9^^,^^10^ We observed similar center-level variation in total costs in Medicare beneficiaries receiving a Watchman device. This finding further strengthens our observation that disparities in patient-level costs are, at least in part, due to differences in practice patterns and service use related to individual centers. Among non-Medicare patients, such variation was even more pronounced, probably due to the superimposed influence of variation in specific insurer-hospital contracts.^11^^,^^12^

We found care at hospitals with low procedural volumes and procedure-related complications independently predictive of high hospital costs. Although our study did not analyze the association between hospital procedural volume and adverse events, an inverse volume-outcome relationship for LAAO procedure has been previously described.^6^ This could be due to variation in operator skills and technical proficiency and management of potential complications at low-volume centers, which may lead to increased resource utilization and thus increased costs. Establishing minimum operator and institutional volume standards for LAAO procedures may potentially result in improved outcomes, thereby reducing unwarranted expenditures.^8^^,^^13^ Additionally, longer hospital stays were associated with high hospital costs. In contemporary practice, patients are hospitalized overnight after LAAO and typically discharged the following day. Same-day discharges have begun to occur but are not widespread. With increasing experience and improving outcomes, same-day discharge in selected patients could be feasible, in doing so reducing length of stay and overall costs.^14^

As the commercial use of percutaneous LAAO with the Watchman device increases in the United States, the wide variability of Watchman-related costs underscores the importance of standardizing periprocedural and hospital practices to achieve high-quality value-based care. Previous cost-effectiveness analyses of the Watchman device have suggested it to be an economically viable stroke risk reduction strategy.^4^^,^^5^ However, such analyses are highly dependent on input variables. The median hospitalization cost in our study was substantially higher than the cost inputs used in prior analyses ($24,500 vs. $16,800), suggesting Watchman implantation could be less cost-effective than previously thought. Therefore, addressing unwarranted cost variation could improve the cost-effectiveness of the Watchman device, further expanding the economic appropriateness of this procedure to a wider population.

### Limitations

To our knowledge, this is the first study to explore center-level differences in cost for LAAO with the Watchman device. In this analysis, we used a large administrative database to evaluate costs. A strength of the present analysis was the use of cost data rather than charges to better reflect the cost of the services being provided. These data are an underestimate of true total costs as they do not account for physician professional fees, line item costs, costs relevant to the family and society, such as transportation to the hospital, and loss of income and productivity if it is necessary to take time off from the workplace. The NIS Database does not track hospitals across years, thereby preventing examination of changes in cost variation at individual hospitals over the study period. Although administrative data sources contain valuable resource utilization information, they lack data on procedural details, medication regimens, costs associated with specific phases of care (Watchman device price, operating room time, anesthesia, intensive care unit stay, etc.). It is possible that certain unmeasured confounders may be present, such as utilization of preprocedural computed tomography; however, we were able to adjust for important factors demonstrated to affect resource utilization. It is possible that coding errors may exist in administrative data sources. The current analysis utilized data from 2016 to 2018, during which Watchman 2.5 device was utilized for LAAO. The Watchman 2.5 device is associated with higher rates of complications compared to the Watchman FLX device approved in 2020.^15^ As shown in our study, periprocedural complications contributed independently to increased costs, and therefore contemporary utilization of the Watchman FLX device may mitigate certain costs related to device associated complications like pericardial effusion requiring intervention and associated prolonged hospitalization. Nevertheless, our findings demonstrate considerable variability in patient costs for elective LAAO attributable to the hospital and emphasize the need for further studies analyzing cost variation related to implantation of more contemporary LAAO devices to develop targeted cost containment strategies. Furthermore, given the retrospective nature of the study and the limitations of the database, we are unable to ascertain the association between cost and complications.

## Conclusions

In this national-level analysis of hospital costs in patients undergoing LAAO with the Watchman device, we found significant variation from center to center even after accounting for important patient factors, hospital volume and location, and length of stay. As the burden of AF increases and a greater number of institutions utilize this therapy, examination of factors associated with hospital-based variation is warranted to develop value-based health care systems.

## Ethics Statement

This study and the research reported adheres to the relevant ethical guidelines.

## Funding

The authors have no funding to report.

## Disclosure Statement

The authors report no conflict of interest.
