# Supplementary material for: Center-Related Variation in Hospitalization Cost for Patients Undergoing Percutaneous Left Atrial Appendage Occlusion
Source: Struct Heart. 2024 Oct 24;9(1):100376. doi: 10.1016/j.shj.2024.100376 (PMC11864124; doi:10.1016/j.shj.2024.100376)

**Supplementary Figure 1**: Distribution of Median Cost of Elective LAAO Device Hospitalization Per Center 2016-2018


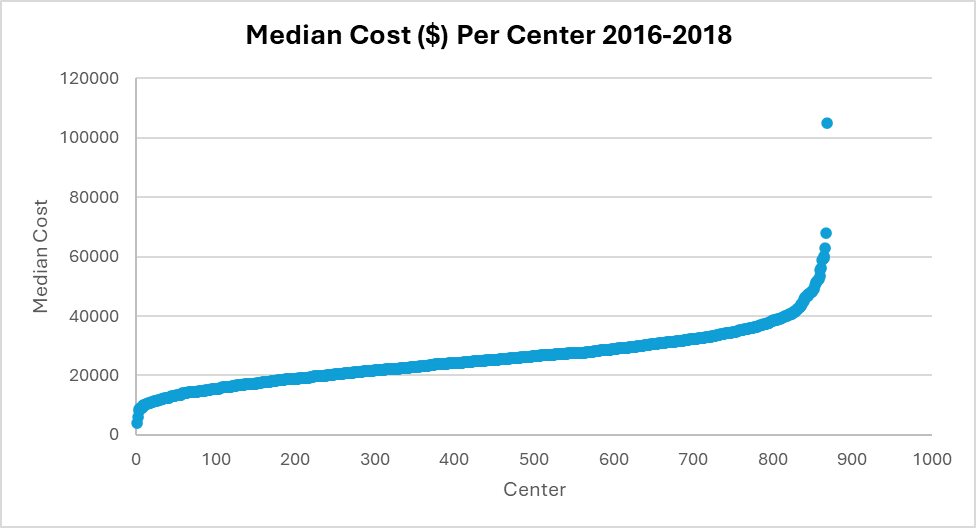


**Supplementary Figure 2:** Distribution of Median Cost of Elective LAAO Device Hospitalization Per Center 2016


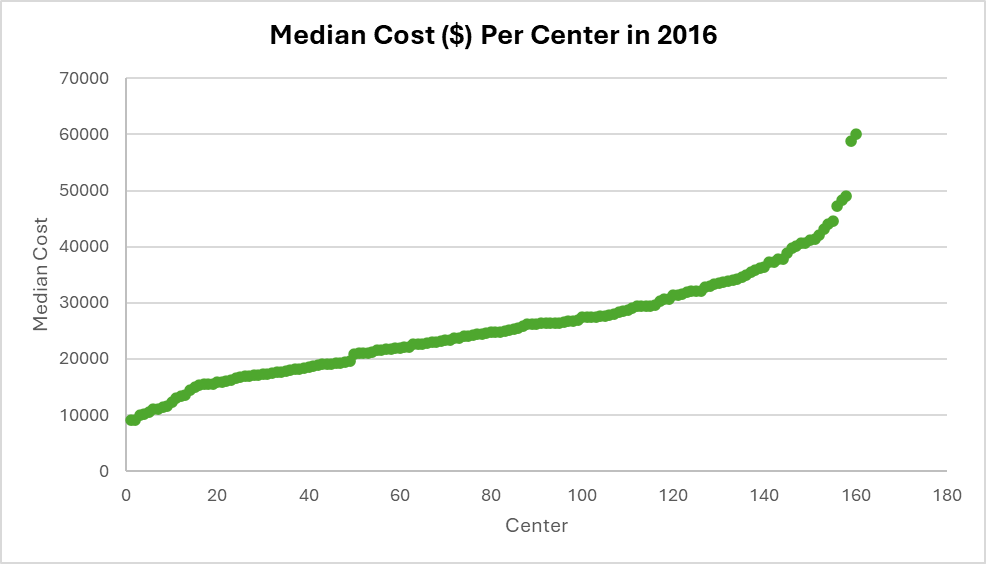


**Supplementary Figure 3:** Distribution of Median Cost of Elective LAAO Device Hospitalization Per Center 2017


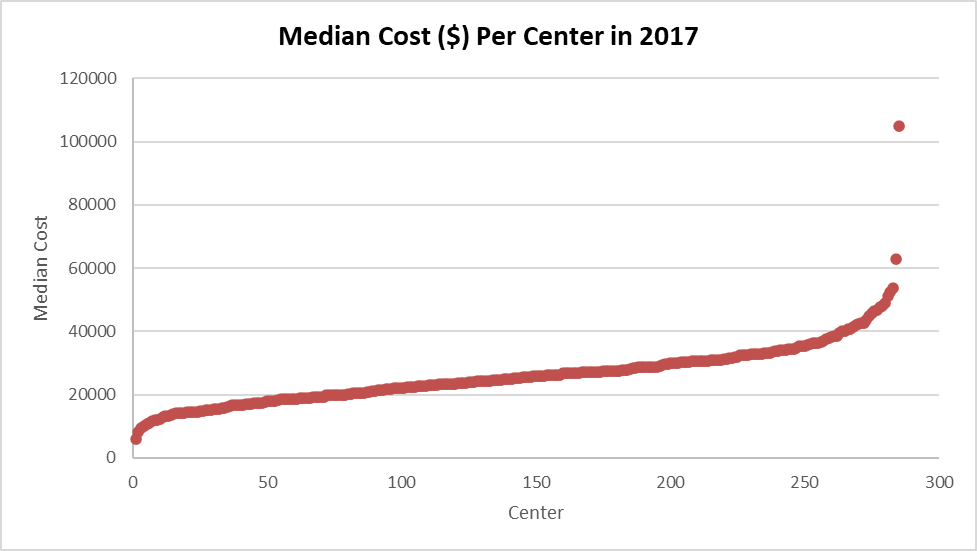


**Supplementary Figure 4:** Distribution of Median Cost of Elective LAAO Device Hospitalization Per Center 2018


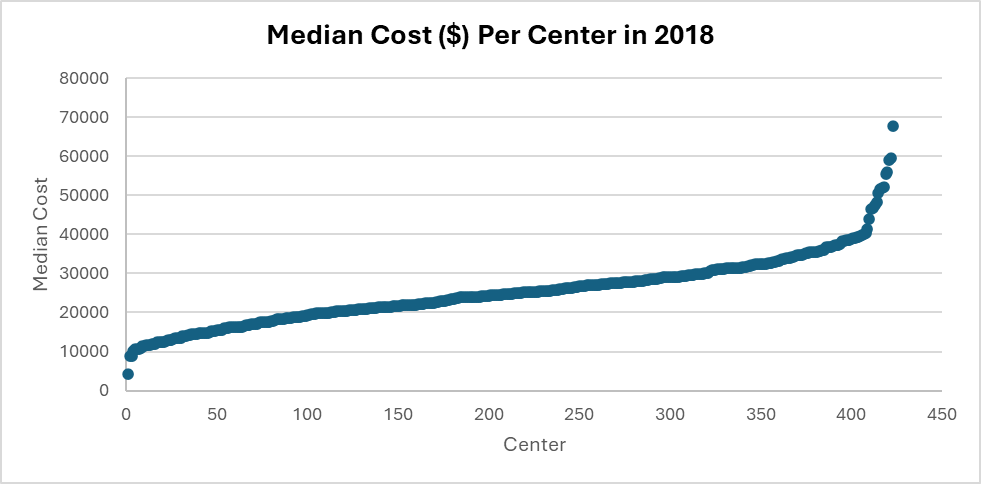


**Supplementary Figure 5:** Positive Skewed Distribution of Total Cost of Hospitalization for elective LAAO Device Implantation 2016-2018


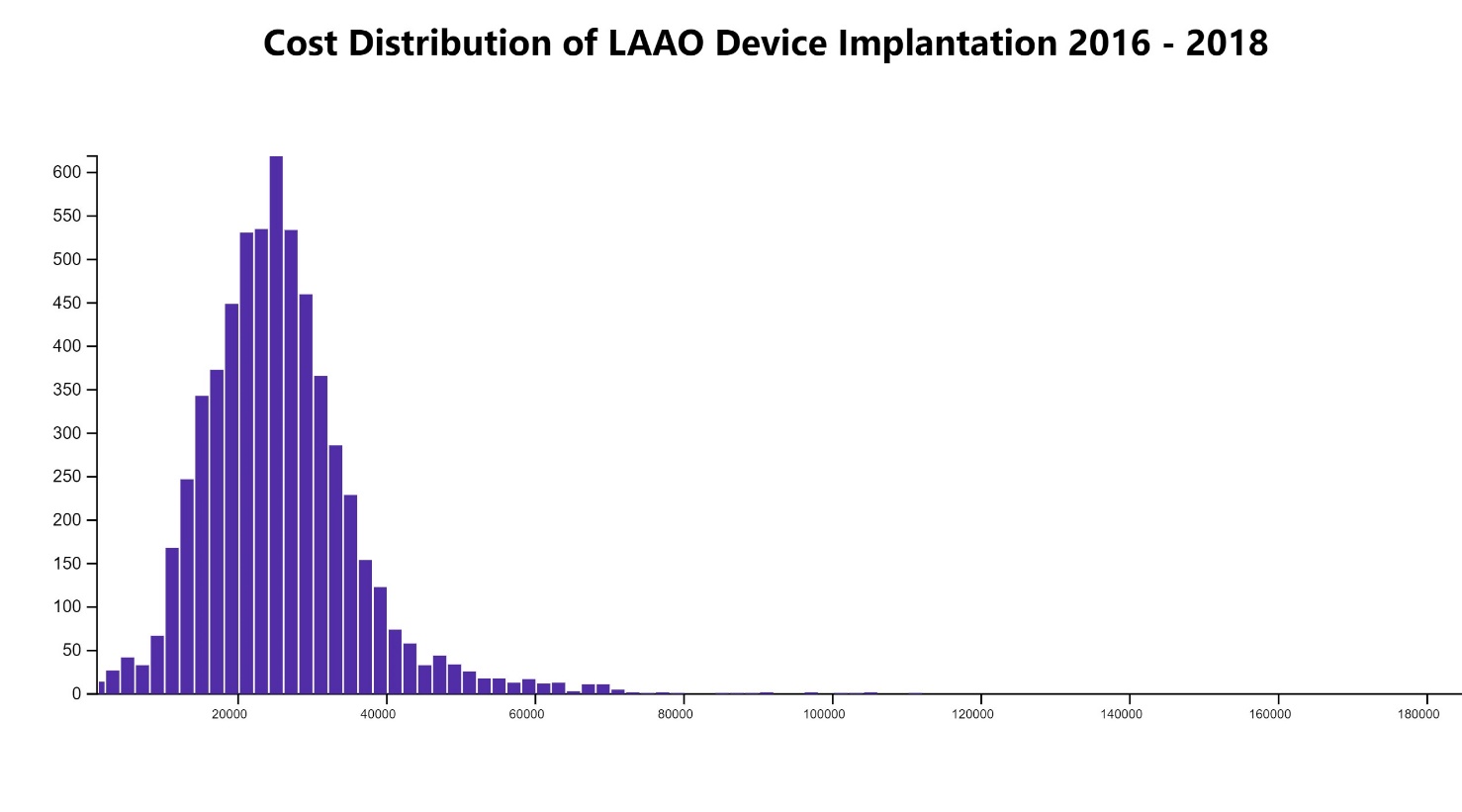

Supplement: Supplementary Figures [file mmc1.docx]
