# Supplementary material for: Center-Related Variation in Hospitalization Cost for Patients Undergoing Percutaneous Left Atrial Appendage Occlusion
Source: Struct Heart. 2024 Oct 24;9(1):100376. doi: 10.1016/j.shj.2024.100376 (PMC11864124; doi:10.1016/j.shj.2024.100376)
Supplement: Supplemental Table 1 [file mmc2.docx]

**Supplementary Table 1:** ICD-10 Codes Used to Define In-hospital Major Adverse Events

| Variable | ICD-10 Code |
| --- | --- |
| Watchman/LAACD | 02L73DK |
| Acute Myocardial Infarction | I21.01, I21.02, I21.09 , I21.11 , I21.19, I21.21, I21.29, I21.3, I21.4 , I21.9 |
| Stroke/TIA | I60, I61, I62, I63, G45.9 |
| Pericardiocentesis or Pericardial surgery | 02CN0ZZ, 02NN0ZZ, 0W9D00Z, 0W9D0ZX, 0W9D0ZZ, 0WCD0ZZ, 0W9C30Z, 0W9C3ZZ, 0W9D30Z, 0W9D3ZX, 0W9D3ZZ, 0W9D40Z, 0W9D4ZX, 0W9D4ZZ, 02N60ZZ, 02N70ZZ, 02NK0ZZ, 02NL0ZZ, 02C60ZZ, 02C70ZZ, 02C80ZZ, 02C90ZZ, 02CK0ZZ, 02CL0ZZ, 02PA0YZ, 02WA0YZ |
| Pericardial Effusion/Tamponade | I31.4, I31.3 |
| Bleeding | D7801, J95830, H59322, G9732, L7602, H9541, D7821, K9161, H59329, G9752, L7622, I97410, E3601, K91840, H9522, H59112, M96811, I97418, G9731, L7601, H9542, H59119, M96831, I97610, G9751,L7621,I97411,H59122,N9962,I97618,H59111,M96810,I9742,H59129,N99821,J9561,H59113,M96830,I97611,H59312,D7801,J95830,H59121,N9961,I9762,H59319,D7821,K9161,H59123,N99820,J9562,H59322,E3601,K91840,H59311,D7802,J95831,H59329,G9731,L7601,H59313,D7822,K9162,H9522,G9751,L7621,H59321,E3602,K91841,H9542,H59111,M96810,H59323,G9732,L7602,I97411,H59113,M96830,H9521,G9752,L7622,I9742,H59121,N9961,H9541,H59112,M96811,I97611,H59123,N99820,I97410,H59119,M96831,I9762,H59311,D62,I97418,H59122,N9962,J9562,H59313,,I97610,H59129,D7802,J95831,H59321,,I97618,H59312,D7822,K9162,H59323 ,J9561,H59319,E3602,K91841,H9521 |
| Blood Transfusion | 30233H0, 30233H1, 30233N0, 30233N1, 30233P0, 30233P1, 30243H0, 30243H1, 30243N0, 30243N1, 30243P0, 30243P1 |
| Systemic Embolization | I74.xx, I75.xx, N28.0, K55.011, K55.021, K55.031, K55.041, K55.051, K55.061 |
| Vascular Complication | I97.410, I97.418, I97.610, I97.618, I97.620, I97.631, I97.638, D62, S09.0xxA, S15.xxxA, S25.xxxA, S35.xxxA, S45.xxxA, S75.xxxA, T11.4, T13.4, I97.51, I97.52, I77.0, T80.1XXA, T81.710A, T81.711A, T81.718A, T81.72XA |
